# Gynecological malignancies at tertiary care hospital, Pakistan: A five-year review

**DOI:** 10.12669/pjms.37.3.3596

**Published:** 2021

**Authors:** Tayyiba Wasim, Javeria Mushtaq, Ahmad Zunair Wasim, Gul-e- Raana

**Affiliations:** 1 Dr. Tayyiba Wasim, FCPS. Department of Gynecology, Services Institute of Medical Sciences, Lahore, Pakistan; 2 Dr. Javeria Mushtaq, FCPS. Department of Gynecology, Services Institute of Medical Sciences, Lahore, Pakistan; 3 Ahmad Zunair Wasim, Student MBBS. Lahore Medical and Dental College, Lahore, Pakistan; 4 Dr. Gul e Raana, FCPS. Department of Gynecology, Services Institute of Medical Sciences, Lahore, Pakistan

**Keywords:** Carcinoma Ovary, Carcinoma endometrium, Cervical cancer, Gynecological malignancies

## Abstract

**Background & Objective:**

Gynecological malignancies are important cause of female morbidity and mortality. They pose significant burden on health resources in low middle-income countries. Data on presentation and risk factors can help in early identification and reduce this burden. Our objective was to evaluate frequency, stage of presentation and risk factors of gynecological malignancies in a tertiary care setting.

**Methods:**

It was cross sectional study done in Gynecology Department, Services Institute of Medical Sciences, Services Hospital, Lahore from January 2015- December 2019. The records of the patients were retrospectively reviewed to include all cases of gynecologic malignancies. Demographic information, frequency, risk factors, symptoms, grade and stage of tumor was collected.

**Results:**

There were 122 patients diagnosed with gynecological malignancy during the study period. Ovarian cancer was seen in 60 (49.18%) patients followed by cervical cancer in 29(23.7%), endometrial cancer 27(22.1%) and vulva 06(4.9%). Mean age for all cancers was 51±12.7 to 55±9.3 except cervical cancer which was seen in 43±8.9 years. Patients with ovarian cancer had significantly more hypertension and diabetes (p<0.05). Heavy menstrual bleeding and postmenopausal bleeding was significantly seen in patients of endometrial and cervical cancer (p<0.05). Abdominal symptoms of pain, mass and distension were seen in patients with ovarian cancer (p<0.05). Majority patients presented in advanced stage. Among ovarian cancer, 52/60(86.6%) were epithelial in origin while 25(86.2%) cervical cancer and all vulva cancers were squamous cell carcinoma.

**Conclusion:**

Ovarian cancer was commonest gynecological malignancy followed by cervical cancer. Late presentation with advanced stage was seen in majority of all cancers.

## INTRODUCTION

New global cancer data suggests that cancer burden has risen to 18 million cases and 9.6 million deaths per year with more than half of the cases residing in Asia.^1^ According to World Health Organization, cancer is second leading cause of death less than 70 years in 92/172 countries.^2^ The diagnosis of malignancy is devastating for the patient not only in terms of anguish that it brings but also mortality and morbidity associated with it. Financial burden to the family and state is another worrisome problem. Gynecological malignancies pose a significant problem to women health. Frequency of these gynecological malignancies is different in different countries depending upon various factors like socioeconomic background, genetic tendency and life style. According to latest figures, breast cancer is the commonest cancer which accounts for 46.3% of cancers in women followed by carcinoma cervix (13.6%), endometrium (8.4%), ovary (6.6%) and vulva in 0.9% of cases.^3^ Incidence of cervical cancer has declined in the developed world with 80% of cases are now being reported from developing countries. The reason being successful cervical cancer screening program by pap smear.

Pakistan has no proper cancer registry although two registries exist with name of Punjab registry and Karachi cancer registry but they are not properly maintained due to lack of resources. Ovarian cancer is the most commonly reported gynecological malignancy from various institution-based studies from Pakistan.^4^,^5^ Studies from India and Bangladesh report cervical cancer as most common malignancy.^6^,^7^ Gynecological cancer is under reported from Pakistan due to various factors. High quality cancer registry and population-based data is needed to estimate burden of disease. It will help generate proper evidence-based control programs for countries where most of the disease resides.

We planned this study to generate local data about the frequency, presentation, stage and risk factors of various gynecological malignancies presenting to tertiary care hospital. It will help devise strategies for early diagnosis, timely management and prioritization of cancer control efforts. This is the first review of gynecological cancer data of five years from Services hospital, Lahore. The objective of this study was to assess frequency, pattern of presentation, risk factors, stage and grade of gynecological malignancies in tertiary care hospital.

## METHODS

A cross sectional study was conducted in Gynecology and Obstetrics Department, Services Institute of Medical Sciences, Services Hospital, Lahore from January 2015 to December 2019 after getting IRB Approval (Ref No. IRB/2019/522/SIMS, Dated March 30, 2019). We retrospectively reviewed the records of the Department of Gynecology OPD and inpatients at SIMS to identify all cases of gynecologic malignancies. All cases diagnosed, referred and treated in the Department of Gynecology with histological diagnosis of malignancy over the past five years were included. Patients with gestational trophoblastic tumors and metastasis to genital organs from other Primary sites were excluded. Detailed Information regarding patient age, parity, various risk factors, presentation, and duration of symptoms, histopathologic subtypes and stage of presentation according to FIGO classification was collected on a pre designed proforma and compared amongst various cancers. Those with incomplete records were excluded from the study. The data was analyzed using SPSS 21.0. Numerical variables like age and age of menarche was measured by mean and standard deviation. Categorical variables like type of malignancy, risk factors, presentation and histologic subtypes were presented as frequency and percentages.

## RESULTS

There were 122 patients diagnosed with gynaecological malignancy during the study period. Ovarian cancer was found to be the commonest malignancy with 60 (49.18%) patients followed by cervical cancer in 29(23.7%), endometrial cancer 27 (22.1%) and vulva 06(4.9%). Mean age for all cancers was 51±12.7 to 55±9.3 except cervical cancer which was seen in 43±8.9 years. Family history of ovarian or breast cancer was seen in 04 and 06 patients of ovarian and endometrial cancer respectively (p<0.05). There was no significant association with parity, early age at marriage, site of residence, education, use of oral contraceptive (OCP), obesity and smoking among different cancer patients (p>0.05). Diabetes was a significant risk factor in patients with ovarian cancer (p<0.05) (Table-I).

**Table-I:** Demographic profile of patients with gynecological malignancies

Demographic Data		Ovarian CA N=60	Endometrial CA N=27	Cervical CA N=29	Vulvar CA N=06	P value
Age		51±12.71	58± 12.32	43±8.98	55±9.309	0.166
Parity	Nulliparous	18 (30%)	06(22.22%)	02(6.89%)	01(16.67%)	0.187
	Para1-5	24 (40%)	11(40.74%)	14(48.27%)	01(16.67%)	
	More than 5	18 (30%)	10(37.03%)	13(44.82%)	04(66.67%)	
Age of marriage	<18yr	13 (21.67%)	06(22.22%)	07(24.13%)	02(33.33%)	0.926
	18-25yr	32 (53.33%)	11(40.74%)	13(44.82%)	02(33.33%)	
	26-30yr	09 (15%)	07(25.92%)	06(20.68%)	02(33.33%)	
	>30yr	06 (10%)	03(11.11%)	03(10.34%)	00(0%)	
Age of menarche		12.5±1.64	13.5±1.28	13.4±1.05	14.3±1.03	0.043
Menopause	Yes	49 (81.67%)	23(85.18%)	07(24.13%)	04(66.6%)	<0.001
	No	11 (18.33%)	04(14.81%)	22(75.86%)	02(33.3%)	
Address	Urban	11 (18.33%)	06(22.22%)	10(34.48%)	03(50%)	0.420
	Urban slums	17 (28.33%)	08(29.62%)	08(27.58%)	02(33.33%)	
	Rural	32 (53.33%)	13(48.14%)	11 (37.93%)	01(16.67%)	
Education	Illiterate					0.727
	Elementary /Matric	38 (63.33%) 16 (26.67%)	15(55.56%) 09(33.33%)	13(44.82%) 10(34.48%)	03(50%) 02(33.33%)	
	FA/Graduate	06 (10%)	03(11.11%)	06(20.68%)	01(16.67%)	0.473
Use of OCPs	Yes	12 (20%)	06(22.22%)	10(34.48%)	01(16.67%)	
	No	48 (80%)	21(77.78%)	19(65.51%)	05(83.33%)	0.016
Family history of cancer	Yes	04 (6.67%)	06(22.22%)	00(0%)	00(0%)	
	No	56 (93.33%)	21(77.78%)	29(100%)	06(100%)	0.972
Treatment taken for infertility	Yes	11 (18.33%)	06(22.22%)	06(20.68%)	01(16.67%)	
	No	49 (81.67%)	21(77.78%)	23(79.31%)	05(83.33%)	0.011
Hypertension	Yes	31 (51.67%)	11(40.74%)	05(17.24%)	04(66.67%)	
	No	29 (48.33%)	16(59.25%)	24(82.75%)	02(33.33%)	<0.001
Diabetes mellitus	Yes	36 (60%)	09(33.34%)	04(13.79%)	03(50%)	
	No	24 (40%)	18(66.67%)	25(86.20%)	03(50%)	0.265
Obesity	Yes	28 (46.67%)	13(48.14%)	09(31.03%)	01(16.67%)	
	No	32 (53.33%)	14 (51.85%)	20(68.96%)	05(83.33%)	0.456
Smoking	Yes	18 (30%)	05(18.51%)	11(37.93%)	02(33.33%)	
	No	42 (70%)	22(81.48%)	18(62.06%)	04(66.67%)	

Regarding symptoms, majority (76.5-83.3%) of the patients were symptomatic at presentation. Heavy menstrual bleeding and postmenopausal bleeding was significantly seen in patients of endometrial and cervical cancer (p<0.05). Abdominal symptoms of pain, mass and distension, loss of appetite and weight loss were significantly present in patients with ovarian cancer (p<0.05) (Table-II).

**Table-II:** Symptoms of patients with gynecological malignancies

Presenting Complaints	Ovarian CA (N=60)	Endometrial CA (N=27)	Cervical CA (N=29)	Vulvar CA (N=06)	P-Value
***Asymptomatic***					
Yes	14 (23.33%)	06(22.22%)	07(24.13%)	01(16.67%)	
No	46 (76.67%)	21(77.78%)	22(75.86%)	05(83.33%)	0.982
***Heavy/irregular menstrual bleeding***					
Yes	22 (36.67%)	19(70.37%)	20(68.96%)	02(33.33%)	
No	38 (63.33%)	08(29.6%)	09(31.03%)	04(66.67%)	0.004
***Post-menopausal bleeding***					
Yes	19 (31.67%)	14(51.85%)	04(13.79%)	01(16.67%)	
No	41(68.33%)	13(48.14%)	25(86.20%)	05(83.33%)	0.018
***Loss of weight/appetite***					
Yes	27 (45%)	09(33.33%)	03(10.34%)	00(0%)	
No	33(55%)	18(66.67%)	26(89.65%)	06(100%)	0.003
***Abdominal mass***					
Yes	37 (61.67%)	00(0%)	00(0%)	00(0%)	
No	23 (38.33%)	27(100%)	29(100%)	06(100%)	<0.001
***Abdominal distension***					
Yes	29 (48.33%)	04(14.81%)	00(0%)	00(0%)	
No	31(51.67%)	23(85.18%)	29(100%)	06(100%)	<0.001
***Abdominal Pain***					
Yes	29 (48.33%)	00(0%)	03(10.34%)	01(16.67%)	
No	31 (51.67%)	27(100%)	26(89.65%)	05(83.33%)	<0.001

Majority of the patients of ovarian and cervical cancer presented beyond stage I, while 59.2% patients with endometrial and 50% patients vulvar cancer presented at stage I (p<0.05). Similarly, ovarian, and cervical cancers presented with high grade lesion in 70% and 82.75%of cases, while majority of endometrial and vulva cancers presented with low grade lesion(p<0.05) (Table-III). Among ovarian cancer, 52/60(86.6%) were epithelial in origin while 25(86.2%) cervical cancer and all vulva cancers were squamous cell carcinoma.

**Table-III:** Stage and Grade of gynecological cancers.

Staging and Grading		Ovarian N=60	Endometrial N=27	Cervical CA N=29	Vulvar CA N=06	P value
Stage of disease	1	19(31.66%)	16(59.25%)	05(17.24%)	03(50%)	
	2	08(13.33%)	04(14.81%)	17(58.62%)	02(33.33%)	<0.001
	3	24(40%)	05(18.51%)	03(10.34%)	01(16.67%)	
	4	09(15%)	02(7.40%)	04(13.79%)	00(0%)	
Grade of disease	Low	18(30%)	13(48.14%)	05(17.24%)	04(66.67%)	0.024
	High	42(70%)	14(51.85%)	24(82.75%)	02(33.33%)	

## DISCUSSION

All over the world significant contribution of female morbidity and mortality is attributed to gynecological malignancies. The incidence is much higher in low income countries than developed countries. Ovarian cancer is the second most common gynecological malignancy in USA and Western Europe and accounts for most cancer deaths.^8^ In our study, ovarian cancer was the commonest cancer accounting for 60 (53.5%) patients. Studies from Pakistan have reported ovarian cancer to be more prevalent than other gynecological cancers.^4^,^5^,^9^,^10^ It is also reported to be the commonest cancer in a study from Iran.^11^ It is in contrast to other studies from India and Africa which report cervical cancer to be the commonest cancer.^6^,^7^,^12^,^13^ One reason may be that we have no screening program for cervical cancer but our religious beliefs with monogamous relationship and fewer sexual partners may also be responsible. Uterine cancer was the third most common malignancies after ovary and cervix in our study, seen in 27(22.1%) patients. Prevalence of 11-14% have been reported from other studies of LMIC.^9^-^12^ Studies from developed countries report a higher incidence and mortality of uterine cancer, labelling it fourth common cancer after breast, lung and colorectal cancer in females.^2^,^3^ Regional variations in the incidence may be attributed to genetic, environmental, dietary and socioeconomic factors. We had six patients of vulvar cancer with frequency of 4.9%. Worldwide, vulvar cancer contribute to 7.0% of all gynecological malignancies with higher incidence in UK and Europe.^3^,^8^

In our study, mean age of patients having ovarian cancer was 51±12.71 years, 42 (70%) being multipara with family history in only04(6%) patients. Other studies report peak age of 50-59 years for ovarian cancer.^5^,^6^,^7^,^11^ Efforts have been directed to early detection by devising different screening modalities like risk factor stratification, serial transvaginal scans and tumor markers. It is suggested that screening should be offered to 50 years and above women considering them a high risk group. The patients with cervical cancer were young with peak age of 43±8.98 years. Other studies also report younger age with peak age of at 41-50 years affected.^6^,^7^,^12^,^13^ It is indeed sad that despite being a preventable cancer so many young women are dying from it. The women with uterine cancer were of 58±12.32 years of age in our study whereas data from West report uterine cancer in older women of 70-75 years.^8^,^14^ Increased life expectancy along with use of hormone replacement therapy (HRT) might be the reason of developing uterine cancer at late age in developed countries. The patients of vulva cancer in our study were also of younger age group of 55±9.3 years as compared to older age of 70±4.2 years from developed World.^2^,^3^,^8^ The younger age presentation suggest HPV infection and emphasize need for screening and awareness regarding sexually transmitted diseases(STDs).

In our study, we compared various risk factors amongst all cancers. Various risk factors for ovarian cancer are reported like nulliparity, endometriosis, obesity, treatment of infertility with ovulation induction drugs, oral contraceptives and smoking.^15^,^16^ Early age of menarche 12.5±1.64 was significantly associated with ovarian cancer while significantly more women presented after menopause in patients with ovarian and endometrial cancer. Menopausal women should be considered a high risk group regarding gynecological malignancy as is shown by our study. These women are shy to come to hospital and neglect their issues considering it part of getting old. Separate clinics should be set up to address their issues and counselling regarding symptoms of malignancy should be done. Family history of breast, ovarian and colorectal cancer is considered an important risk factor for patients of ovarian cancer.^17^ In our study, only 04(6.6%) patients had positive family history. Research at molecular and genetic level should be carried out in our population to know the etiology of gynecological cancers so that preventive strategies can be devised.

Risk factors of poverty, living in slums, sexually activity at early age, multiple sexual partners and smoking are reported for cervical cancer.^12^,^13^ In our study, no significant association was found as related to all these risk factors. History of multiple sexual partners could not be elicited as patients were either unaware of husband partners or were reluctant to discuss. Majority of our patients of all cancers were illiterate, belonged to lower socioeconomic strata and resided in rural areas. This is true of all studies from lower middle income countries (LMIC).^9^-^13^ Hypertension and diabetes was significantly associated with ovarian cancer in our study although it is not reported as a risk factor in other studies.^15^,^16^ Diabetes is rampant in South Asia; more studies are needed to confirm diabetes as a risk factor for ovarian malignancy. Increased body mass index(BMI), nulliparity and use of HRT are identified as strong risk factors for uterine cancer.^18^ Obesity was not found a significant risk factor in any of the cancers in our study.

The patients with cervical and uterine cancer presented with menstrual irregularities while patients with ovarian malignancy had abdominal symptoms in majority of cases. The complaint of irregular bleeding was present in patients with cervical cancer for months but diagnosis was delayed. Local doctors treated their symptoms with hormones without actually examining the patient. Proper history and examination are the most important tools to early diagnosis. It is reported that more than 70% patients of cervical cancer in Pakistan, present at advanced stage of malignancy and this is the reason of high mortality.^19^ Established screening program, public awareness regarding sexually transmitted disease, diagnosis and treatment of preinvasive disease has resulted in eradication of cervical cancer from developed world. Lack of knowledge of screening and prevention is an important risk factor in our country. Sultana et al found that only 6.5% of university students and 5% of educated urban class were aware of the causes and screening of cervical cancer.^20^ It is one of the few cancers where definite and treatable premalignant stage is identified with slow progression to malignancy over a period of few years. Screening program with pap smear is not an ideal program for developing world. It is expensive, cytologist dependent and needs a follow up which is not possible in resource poor countries. Cost effective screening program like visual inspection with acetic acid with see and treat approach is recommended by American Society of Clinical Oncology(ASCO) guidelines for basic settings.^21^ Visual inspection with acetic acid is a simple easy approach with good sensitivity and high negative predictive value and should be made a part of national screening program in our country.

Postmenopausal bleeding was major symptom in patients with uterine cancer. Symptoms of abdominal pain, mass and distension abdomen along with anorexia and weight loss were significantly seen in patients with ovarian cancer in our study. These abdominal symptoms are also reported from other studies.^9^-^13^ A big dilemma with ovarian cancer is nonspecific symptoms which do not relate to genital tract, moreover, these are late symptoms when the disease is already advanced.^22^ Ovarian cancer is the most lethal gynaecological cancer often called silent killer due to lack of specific symptoms with poor prognosis. The patients with CA vulva came with ulcerative lesion on vulva in 5/6 (83.3%) patients. If detected at early stage, conservative surgery with preserved psychosexual outcome is possible in addition to reduced morbidity and mortality.

In our study, 68.3% of ovarian cancer, 82.7% cervical cancers, 50% cancer vulva and 40.7% endometrial cancer presented beyond stage I. Survival rates are related to stage with 90% survival in stage I and dropping to 20% in stage IV. Late presentation is a dilemma in developing countries as the patient is no more amenable to surgery, which not only affects the survival but also adds cost in terms of chemotherapy and radiation. Radiation therapy is scarcely available, expensive with shortage of trained personnel. There is dearth of specialized oncology centers, a structured screening program and access of women to health care facilities. The ratio of medical oncologist to patient is reported to be 0.027 per 100,000 populations with only 21.4% population having access to radiotherapy services in Pakistan.^23^ There are various socioeconomic and cultural factors associated in addition to lack of resources in low middle income countries.^24^ Poverty has a major role to play and detrimental health impact of growing in a poor family is potentiated by false cultural beliefs they harbor. The constraints of finances and lack of knowledge leads to delay at the hands of local quacks and spiritual healers. Advanced stage cancer has added burden of palliative care which is an essential pillar of cancer care. In addition to pain relief, it involves physical and psychological support to the dying patient which again adds burden to already compromised resources.

Public awareness strategies should be devised by government with focus on screening and early reporting. National cancer registries should be maintained with funds dedicated for research. A late stage presentation and diagnosis can be tackled by developing multiple specialized cancer care centers that are well equipped and provide an affordable treatment to poor masses. All this can help in reduction of mortality and morbidity from these gynecological malignancies in developing country like Pakistan.

## CONCLUSION

Ovarian cancer is commonest gynecological malignancy followed by cervical cancer. Late presentation with advanced stage was seen in majority of all cancers. Public awareness should be created for early presentation to reduce morbidity and mortality.

### Authors’ Contribution:

**TW** conceptualized and designed the study, reviewed the manuscript and approved the final version. He is also the responsible for this study.

**JM** contributed to initial writing of script.

**AZW** did the data entry and statistical analysis.

**GR** maintained data base.
